# Sequencing and comparative genomics analysis in *Senecio scandens* Buch.-Ham. Ex D. Don, based on full-length cDNA library

**DOI:** 10.1080/13102818.2014.956461

**Published:** 2014-10-28

**Authors:** Gang Qian, Junjiao Ping, Zhen Zhang, Delin Xu

**Affiliations:** ^a^ Department of Cell Biology and Genetics, Zunyi Medical College, Zunyi, Guizhou, P.R. China

**Keywords:** *Senecio scandens* Buch.-Ham. ex D. Don, comparative genetics, expressed sequence tags (ESTs), full-length cDNA library

## Abstract

*Senecio scandens* Buch.-Ham. ex D. Don, an important antibacterial source of Chinese traditional medicine, has a widespread distribution in a few ecological habitats of China. We generated a full-length complementary DNA (cDNA) library from a sample of elite individuals with superior antibacterial properties, with satisfactory parameters such as library storage (4.30 × 10^6^ CFU), efficiency of titre (1.30 × 10^6^ CFU/mL), transformation efficiency (96.35%), full-length ratio (64.00%) and redundancy ratio (3.28%). The BLASTN search revealed the facile formation of counterparts between the experimental sample and *Arabidopsis thaliana* in view of high-homology cDNA sequence (90.79%) with *e*-values <1*e* – 50. Sequence similarities to known proteins indicate that the entire sequences of the full-length cDNA clones consist of the major of functional genes identified by a large set of microarray data from the present experimental material. For other Compositae species, a large set of full-length cDNA clones reported in the present article will serve as a useful resource to facilitate further research on the transferability of expressed sequence tag-derived simple sequence repeats (EST-SSR) development, comparative genomics and novel transcript profiles.

## Introduction

Complementary DNA (cDNA) libraries are widely acknowledged as an effective tool for research on gene structure, function and manipulation.[1] Expressed sequence tags (ESTs), being 5′- or 3′-end single-pass-sequenced portions of randomly isolated cDNA clones, represent part of the transcribed region of the genome in given conditions.[2,3] The ESTs obtained from the construction of cDNA libraries have played a crucial role in functional genomics research, e.g. in new functional gene discovery.[4] In many organisms, ESTs have proved useful for the annotation of genes during genome sequencing efforts, for comparative genome studies and for the production of genetic linkage maps.[5,6] In these data analyses, genome annotation is one of the most fundamental and indispensable steps, directly affecting further studies such as molecular evolutionary analyses, transposon tagging and microarray experiments.[7] Moreover, ESTs can provide a powerful resource of sequences that can aid the discovery of novel genes, genome annotation and comparative genomics studies,[8] as well as an overall scan of transcripts involved in organ or tissue development.[9]

The construction and analysis of cDNA libraries has, in recent years, grown to become an indispensable approach in functional genomics analysis, since they are a source of much more detailed information on the genomic mechanisms underlying diverse processes in different organisms.[10] The vast amount of sequence data, including whole-genome sequences, novel transcript profiles, proteome or metabolic information, has expanded our understanding of genomic structures, evolution, gene discovery or gene functions, etc.[11] Development of full-length cDNA collections is one of the effective strategies for increasing the catalogue of gene transcripts. These data serve as a valuable resource to describe gene expression profiles and ultimately classify genes into families based on their functions.[12] Therefore, inclusion of the entire sequence data paves the way for subsequent functional assays such as transcriptome and genome annotation and protein expression analysis [13] for the further study of important genes responsible for phenotypic features and pharmacological characteristics within Compositae species.

*Senecio scandens* Buch.-Ham. ex D. Don, a plant predominantly native to China, plays an important role in Chinese traditional medicine owing to its antibacterial properties. That is why, to facilitate breeding, gene discovery or industrial applications, the plant's characteristics should be studied at the molecular level.[14] However, to the best of our knowledge, at the time our study was initiated, there were few reports on the molecular biology of *S. scandens*. Here, we complement the molecular data presented in our recent report [14] with an experiment on a full-length-enriched cDNA library to obtain further information on novel transcript profiles and functional genomics, using a sample of elite individuals with superior antibacterial properties.

## Materials and methods

### Plant materials

A series of standardization bacteria (*Staphylococcus aureus*, *Pseudomonas aeruginosa*, *Escherichia coli*, *Salmonella paratyphi*, *Shigella flexneri*, *Aeromonas sobria* and *Edwardsiella tarda*) were applied to detect the antibacterial activity of the present experimental materials, according to the methods of Shapiro and Baneyx[15]. As a result of this screening, a sample of elite individuals (SC-32) with superior antibacterial properties was selected to construct a full-length cDNA library.

### Construction of full-length cDNA library

We generated a full-length cDNA library from the elite antibacterial accession, using Creator SMART (Switching Mechanism at 5′ end of RNA Transcript) Construction Kit (CLONTECH) according to the manufacturer's suggestions, as described previously.[14] Briefly, the ligation product (5 μL) of the resultant double-strand cDNA and the vector pDNR-LIB was transferred to XL1-Blue electrocompetent cells (25 μL). The method of consecutive dilution was used to titre the bacteria solution, as described by Abe et al. [12]. Then, colony counts were obtained from overnight culture plates with a Luria-Bertani (LB) medium inoculated with the diluted solution (1 mL). The titre was calculated according to the following formula: colony × 10^3^ × 10^3^ colony-forming units (CFU/mL).

### Sequence data trimming, assembly and annotation

The plasmid DNA of each clone was directly prepared from bacterial cultures of a glycerol stock plate by the linear rolling circle amplification (RCA) method [16] using a TempliPhi HT DNA amplification kit (GE Healthcare, UK). End sequencing of 10,000 clones was carried out with an iCycler iQ SYBR Green polymerase chain reaction (PCR) (BIO-RAD Co., LTD., America) using an M13 sense and antisense primer. Raw sequence data (chromatograms) were base-called using the Phred program [17] and vector sequences were then detected by using cross-match. The low-quality region (Phred quality score <20, and >20 bases repeated) was discarded. We trimmed off the vector sequences of both ends of each read, using the sim4 program.[18] Sequences data of lengths shorter than 100 bases after the trimming process were also omitted from further analysis. In addition, we excluded such sequences of the repetition of a single nucleotide in a sequence longer than 10.00% of its total length. The ESTs were assembled using the CAP3 program with a 40 bp overlap and 90.00% sequence identity, as described by Huang and Madan [19].

### Sequence analysis

DNA sequences generated from each cDNA clone were carefully edited to remove the vector sequence and the low-quality 3′ sequence. Generally, ESTs longer than 150 bp and containing ambiguity of less than 4.00% were considered useful for data analysis.[20] To obtain a non-redundant (nr) set of transcripts, we clustered 5′- or 3′-end sequences according to clone names in the CAP3 output. The ‘.ace’ file and the ‘.singlets’ from the CAP3 output were parsed to build scaffolds, which were clusters of sequences representing a unique transcript for which the positional relation and direction of the fragments was implied. As a means to estimate the similarity to genes from other plants, we aligned the above sequences with known information, using a BLASTX search (*e*-value < 1*e* – 5) against protein data sets from TAIR (http://www.Arabidopsis.org) and clusters of orthologous groups (COGs), as described by Rhee et al. [21] and Tatusov et al. [22]. These ESTs were translated into six reading frames and searched against the nr peptide database at the National Center for Biotechnology Information (NCBI) (http://www.ncbi.nlm.nih.gov), using BLASTX Version 2.2.9.[23] Multiple sequence alignment between the amino acid sequences of candidate clones and their homologues of the other species were also analysed by using CLUSTAL W.[24] For the detection of novel genes in the experimental accession, UniGene cluster data were applied to carry out the putative coding sequences in GenBank for the BLASTN analysis.

## Results and discussion

### Quality of the full-length cDNA library

**Figure 1.**
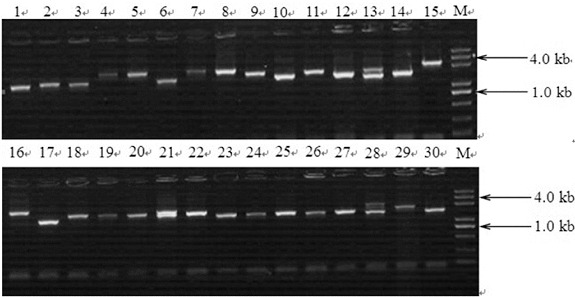
Insert length of the cDNA library. Lane 1–30: randomly selected cDNA clones from the library were used as templates for PCR amplification; Lane M: 5 kb DNA ladder.

We used the sample of elite individuals with superior antibacterial properties to generate a full-length-enriched cDNA library in *S. scandens*. The 30 clones randomly selected from this library were examined to evaluate the quality of this library. As shown in Figure 1, the insert size was distributed from 1000 to 4000 bp, with the average size 1.70 Kb of the positive fragment. The satisfactory quality of our primary cDNA library was obtained, involving the parameters of library storage (4.30 × 10^6^ CFU), efficiency of titre (1.30 × 10^6^ CFU/mL), transformation efficiency (96.35%), full-length ratio (64.00%) and redundancy ratio (3.28%). The quality of our library was then submitted for further sequence analysis because it contained a lot of full-length inserts and was shown to meet the necessary criteria. Our previous study [14] presents limited data on the cDNA library construction, based on screening of the most contrasting phenotypic accessions on antibacterial characteristics. Here, a comparative genomics analysis is presented, as well as further analysis of these functional sequences. The ultimate purpose is to develop and enhance such resources that will support and accelerate many molecular biological aspects of further research across the Compositae species, as described below.

### Features of full-length sequencing data

**Figure 2.**
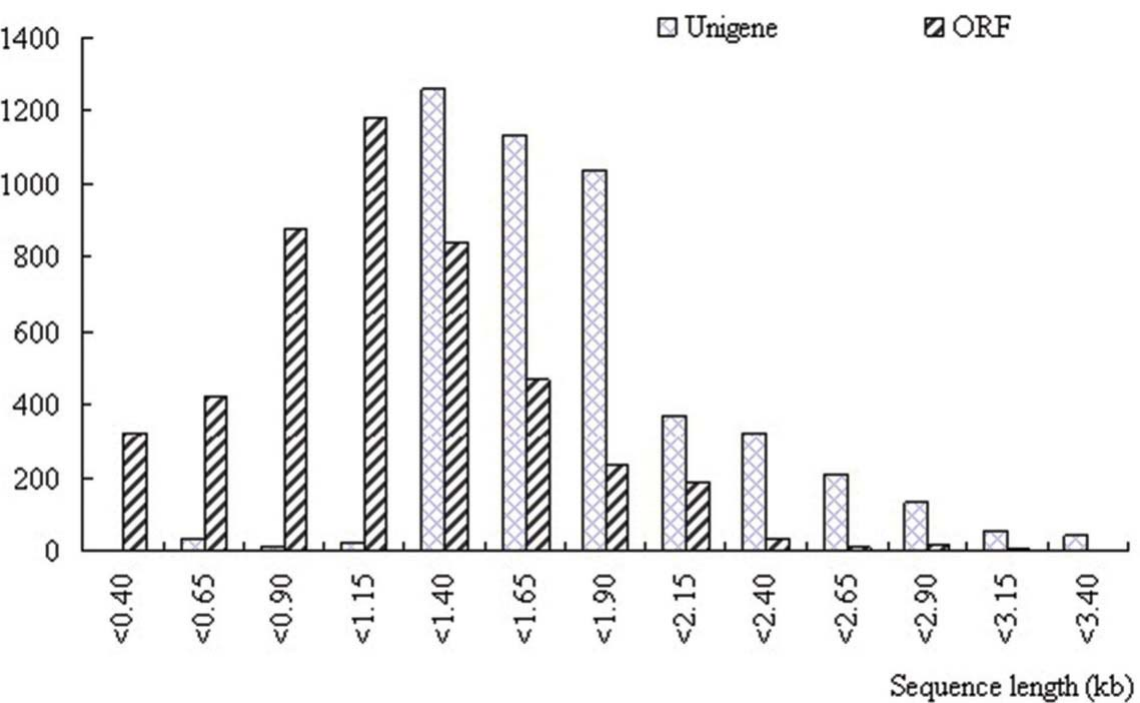
Length distributions of unigenes and ORFs. Data obtained from a total of 4608 full-length sequences.

We collected 3712 plasmid clones from each fraction, resulting in a total of 11,403 clones, and successfully sequenced 8072 clones from the 3′ region of the cDNAs. After eliminating redundant clones, we obtained a total of 5796 nr clones. Although our library was not normalized, it successfully generated the genetic sequence data of cDNAs, suggesting that it could be considered a novel resource for obtaining information from nr genomic sequences. Following the common approach, clones which had both read sequences showing significant sequence similarity to known proteins were analysed to confirm whether they contained initiation codons and poly (A)^+^ tails.[25] Our results indicated that the entire sequences of the full-length cDNA clones will be extremely informative, as they consist of the major part of the functional genes identified by a large set of microarray data from the present experimental material. Length distributions of unigenes and open reading frames (ORFs) from the present sample cDNA sequencing data are shown in Figure 2. The satisfactory size of cDNA inserts could be considered to serve as evidence that our data cover a wide range of ORFs. That is possible because a full-length cDNA represents a single splice variant from each transcription unit. Thus, our results are in line with the report of Umezawa et al. [25], who successfully generated a soybean cDNA library that captured a wide range of cDNA inserts without any bias.

A full-length-enriched cDNA library is properly constructed if it includes a high proportion of full-length cDNA clones and keeps complete coding regions, splicing information and 5′- and 3′-UTR (untranslated region) sequences.[25] The fact that most clones contain the 5′- and 3′-UTRs in addition to the complete coding sequences is a major advantage of this approach.[13,25] In future studies, the 5′-UTR data can help in the identification of promoter sequences, and the full-length sequences would make it possible to search for orthologues/paralogs or gene families in the genome of *S. scandens*. This collection will also be useful for analysis of gene expression profiles and functions in plants.[25] From this point of view, the obtained sequencing data may help in the construction of genetically complex pools of RNA data to facilitate novel gene discovery and reduce overall redundancy. Therefore, a lot of information of genomic resources will need to be collected to understand the interaction of metabolic systems within the Compositae species.

### Comparative genomics and bioinformatics analysis

**Figure 3.**
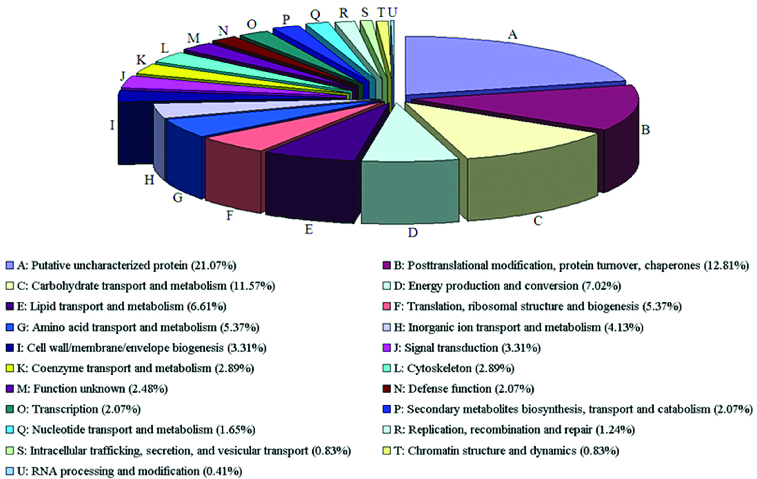
Functional classification of sequences with the homologues in the BLASTX analyses (*e*-values < 0.001).

A comparative analysis of sequences was performed between *S. scandens* and *Arabidopsis thaliana*, with a large body of functional information and expression data for each gene. Based on the specific functional categories in *S. scandens*, putative uncharacterized proteins (21.07%) might be derived from ‘molecular function unknown’ clones or rare transcripts using a classification system designed by the Gene Ontology Consortium (http://www.geneontology.org) (Figure 3). As mentioned above, it makes hereby sense that the probability for novel cDNA clones to appear in this library is determined by comparative genomics and by their expression levels. Therefore, the obtained sequencing data will serve as a valuable resource to help describe novel gene expression profiles, classify genes and aid the precise annotation of the genomes of the Compositae plants. Our results are in parallel with the general assumption that, when large-scale ESTs are generated, a useful library should comprise at least 50% new genes, a broad variety of transcripts and no more than 20% uninformative sequences.[1,26]

To understand the species-specific metabolism mechanisms, it is important to make a comparison with the changes in gene expression of *Arabidopsis*. For this purpose, we compared 5796 nr sequences with the all-genes database (www.allgenes.org), and found that 162 had no match in the database. The BLASTN search revealed the facile formation of counterparts between *S. scandens* and *A. thaliana* in view of high-homology cDNA sequences (90.79%) with *e*-values <1*e* – 50. We also found 23 clones with homology to genes encoding proteins from species other than *Arabidopsis* (see Table S1 in the online supplementary appendix).

To further observe the characteristics of full-length sequences, we determined the distribution of all sequenced scaffolds involving nr EST sequences by clustering the CAP3 assembly data. These analyses show that full-length cDNAs are a useful tool in functional classification of sequences with homologues and in detailed analysis of expression patterns of single transcripts. The analysis of data by cellular components indicates that, in *S. scandens*, a higher frequency of clones related to plastids and unknown cellular component was detected (Figure 4(A)). For molecular function analysis, gene ontology searches showed higher transcript frequency of those genes that encode structural molecules and nucleotide-binding activities in *S. scandens* than in *A. thaliana* (Figure 4(B)). As compared with that of *Arabidopsis*, a higher frequency of the clones related to unknown metabolic processes was observed in the experimental sample (Figure 4(C)), suggesting that sets of metabolism-related genes representing pharmacological traits possibly remain unclear. These results are in good accordance with the report of Nanjo et al. [27] on the full-length enriched ESTs of *Populus nigra*. Figure 4. Clustering and functional annotation of full-length cDNA clones in *S. scandens*, using the *Arabidopsis* gene ontology database: cellular component (A), molecular function (B) and biological process (C).

**Figure:**
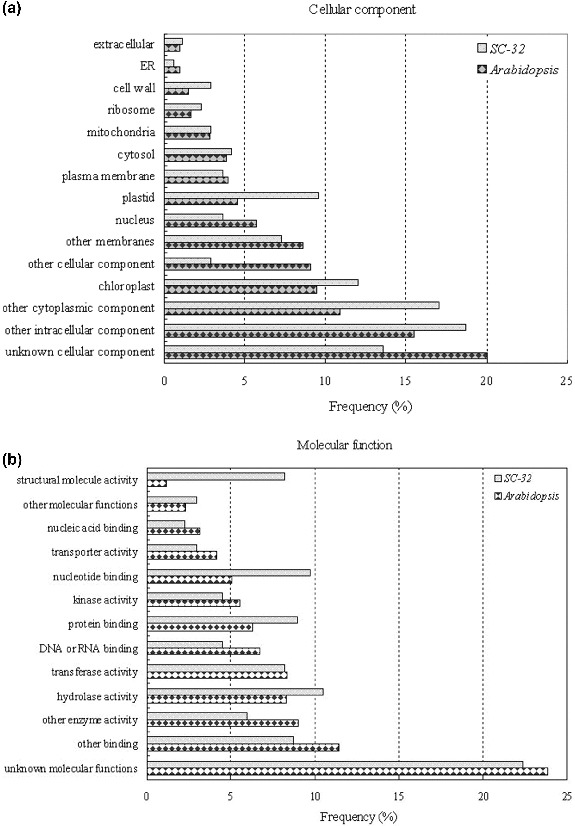

**Figure:**
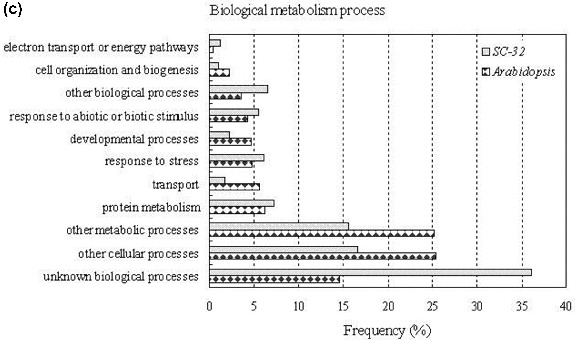


### Genome resource and its potential usefulness

As a useful tool for functional genomics studies on Chinese traditional medicinal plants, our sequencing data are a valuable resource for the distribution of gene ontology annotations for mRNAs from other Compositae plants, and were deposited to NCBI with GenBank accession numbers JK784523–JK784613, JK820361–JK820513, KC149908.1, KF887495, KF887496, etc. Owing to the recent developments in functional genomics, a growing number of ESTs are being deposited in public sequence databases, thus accumulating a potentially rich resource of molecular markers.[28] When related to the coding regions of the genome, these EST-derived markers could be employed in studies on marker–trait associations.[29] At present, an accurate screening at the molecular level for high-quality biological traits is still difficult to achieve in *S. scandens*, considering its genetic diversity and diverse eco-geographic regions. Based on comparative genomics studies, our results of the full-length cDNA collections can serve as a powerful tool to facilitate genomic or other ‘-omics’ research efficiently in *S. scandens*. Therefore, the sequence analysis in this study will facilitate further research on DNA markers to evaluate the genetic diversity of *S. scandens* and screen for high-quality antibacterial agents.

Due to the codominant and usually single-locus nature of EST-SSR loci, their alleles can be identified in different genotypes of the same species and often in those of other close relatives. That is why, a specific set of simple sequence repeats (SSRs) can be applied in different sets of genotypes or mapping populations, which makes them especially useful for variability analysis, fingerprinting, molecular marker development, marker-assisted selection, map construction and comparative studies.[30] For example, several techniques have been established to prepare full-length cDNA enriched libraries for mapping of the late-bolting characteristic of *Brassica rapa* [31,32] from various organisms,[33] and SSR markers are known to be optimal for plant breeding in various plants such as bread wheat,[34] maize,[35] rice [36] and poplar.[27] In light of these advances, our construction of a full-length cDNA library of *S. scandens* would also be useful for the development of EST-SSRs that can act as versatile data to study genetic variability and plant breeding across Compositae species.

## Conclusions

In this study, we performed an analysis of the full-length-enriched cDNA library of *S. scandens* previously constructed by us using elite material with superior antibacterial properties. The BLASTN search revealed the facile formation of counterparts between our experimental sample and *A. thaliana* in view of high-homology cDNA sequences (90.79%). Sequence similarity to known proteins indicates that the entire sequences of full-length cDNA clones mostly consist of functional genes identified by a large set of microarray data from the present experimental material. Our entire sequences of full-length cDNA clones would be useful in functional genomics analyses for novel transcript profiles and distributions of gene ontology annotations within Compositae species.

## Supplemental data

Supplemental data for this article can be accessed at http://dx.doi.org/10.1080/13102818.2014.956461.
